# Comparative evaluation among different materials to replace soft tissue in oral radiology studies

**DOI:** 10.1590/S1678-77572010000300012

**Published:** 2010

**Authors:** Maria de Paula CALDAS, Flávia Maria de Moraes RAMOS-PEREZ, Solange Maria de ALMEIDA, Francisco HAITER-NETO

**Affiliations:** 1 DDS, MSc, PhD, Department of Oral Diagnosis, Piracicaba Dental School, State University of Campinas, Piracicaba, SP, Brazil.; 2 DDS, MSc, PhD, Associate Professor, Department of Oral Diagnosis, Piracicaba Dental School, State University of Campinas, Piracicaba, SP, Brazil.; 3 DDS, MSc, PhD, Full Professor, Department of Oral Diagnosis, Piracicaba Dental School, State University of Campinas, Piracicaba, SP, Brazil.

**Keywords:** Tissues, Radiography dental, Simulate

## Abstract

**Objective:**

The aim of this study was to establish which materials afford better simulation of soft tissues in Oral Radiology studies.

**Material and Methods:**

The sample was composed of four materials in eleven different thicknesses to simulate the soft tissues of the face. The mean values of the relative amounts of radiographic contrast of the materials were determined and compared to a gold standard value, which was obtained from 20 patients who were referred to have periapical radiographs taken of the left mandibular molars. Data were subjected to statistical analysis with Dunnett's test (p<0.05).

**Results:**

The mean value of the relative amounts of contrast encountered in the patients was 0.47, with a range between 0.36 and 0.64 for all 44 material/thickness combinations. The majority of the tested materials showed values close to those of the patients’ tissues, without statistically significant differences among them. The values of only three materials/ thickness combinations differed statistically from those of the patients’ tissues.

**Conclusions:**

Based on the results of the present study, it may be concluded that except for utility wax (4 mm and 8 mm) and water (4 mm), all materials tested at different thickness could be used as soft tissue substitute materials in Oral Radiology studies.

## INTRODUCTION

In Oral Radiology research, phantoms are frequently used to simulate the patient’s body. There are, however, some requirements for tissue substitutes. A material that could be easily obtained and simulate the soft tissue would be helpful for Oral Radiology professors and researchers. It is important for these materials to be capable of being accurately measured, available, reproducible, and ready to be used in any instance^7^. Thus, standardization would be perfectly possible. The development of phantoms that present densities similar to those observed in patients is very important because it avoids unnecessary radiation exposure. According to the ALARA principle, all unnecessary exposure to radiation should be avoided^15^.

Dry mandibles are widely used in optical bone density studies^3,5,6,10^. However, the studies previously reported in the literature do not consider the significant influence of the patient’s soft tissue located between the bone and the x-ray beam. The intensity of an x-ray beam is reduced by interaction with the matter it encounters. This attenuation results from interactions of individual photons in the beam with atoms in the absorber. The x-ray photons are either absorbed or scattered out of the beam^15^. It is expected that this phenomenon also occurs when the x-ray beam interacts with the mandible. It is thus very important to consider the presence of a specific material that simulates the soft tissue of the human face when obtaining the optical value of the jaw density^3^.

Various materials simulating soft tissues have been cited: water, wax, self-polymerizing resin, paraffin and polyethylene^1-3,5,9,13^ . However, all studies consider bovine muscle as gold standard to test a specific soft tissue substitute material.

A specific substitute tissue that presents contrast values similar to those of humans is essential and might facilitate all radiological experiments and education. Thus, the aim of this study was to establish which materials offer better simulation of soft tissues in Oral Radiology studies, using the human soft tissue as gold standard.

## MATERIAL AND METHODS

**Figure 1:**
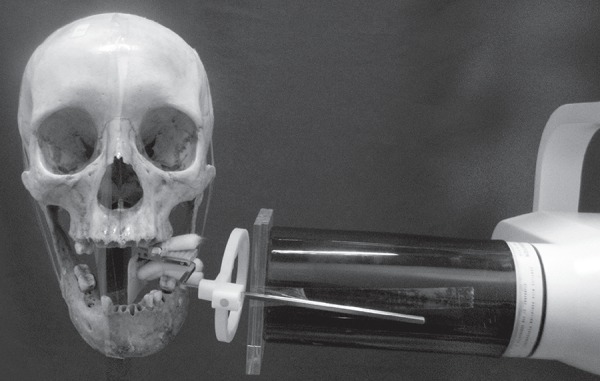
Dental film fixed in a film holder attached to the posterior region of a dry mandible with buccal interposition of acrylic in order to simulate soft tissue

The sample was composed of four different materials in eleven different thickness (4, 8, 12, 15, 20, 24, 28, 32, 36, 40 and 45 mm): self-polymerizing acrylic resin, utility wax, wood and a 2 mm-thick polymethylmethacrylate box filled with a 2-mm-thick water layer. All materials were exposed using InSight dental film (Eastman Kodak Co., Rochester, NY, USA) with the addition of an aluminum step-wedge. Standardized conditions were used: Ge 1000 machine (General electric Co., Milwaukee, WI, USA), operating at 70 kVp, 10 mA, and 40 cm focus-film distance. The dental films were attached to a dry mandible, with buccal interposition of a soft tissue substitute material, and the x-ray beam was projected perpendicular to the film (Figure 1). Three standardized radiographs of each material at its specific thickness were obtained from the mandibular posterior segment of a cadaver.

Using a densitometer (MRA, Ribeirão Preto, SP, Brazil), it was possible to determine the radiographic density value of all materials at their specific thicknesses. This value was used to obtain the relative amounts of contrast or contrast index, according to Price^12^ (1986), using the following equation:

C= D_2_-D_7_/ 0.5(D_2_+ D_7_), where C is the relative amounts of contrast or contrast index, D_2_and D_7_ are the second and seventh step of the density scale, respectively.

Radiographs from 20 patients referred to have periapical radiographs of mandibular left molars taken at the Oral Radiology Clinic of Piracicaba Dental School, State University of Campinas, were used. The research protocol was approved by the Research ethics Committee of Piracicaba Dental School, and the patients signed an informed consent form before their enrollment.

All radiographs were taken in accordance with routine procedures. The patients wore lead aprons and leaded thyroid collars in order to enhance the diagnostic benefits of dental radiographs and minimize patient’s exposure to radiation. All images were exposed with the same Ge 1000 machine, InSight dental film and focus-film distance. Using a densitometer, it was possible to determine the radiographic density value of each patient. This value was used to obtain the soft tissue relative amounts of contrast or contrast index, according to Price^12^ (1986), as mentioned above. The mean of these values was used as gold standard.

All films were processed using a Gendex GXP (Gendex Dental Systems, Lake Zurich, IL, USA) with fresh Kodak processing liquids and operating time of 5 min.

The mean values of the relative amounts of radiographic contrast of each material (at its specific thickness) were compared with the gold standard value using Dunnett’s statistical analysis (p<0.05).

## RESULTS

The results showed that the mean value of the relative amounts of contrast encountered in the patients was 0.47, with a range between 0.36 and 0.64 for all 44 material/thickness combinations. The majority of the tested materials showed values close to those of the patients’ tissues, without statistically significant differences among them. It was possible to observe that the values of only three materials/ thickness combinations differed statistically from those of the patients’ tissues, utility wax (4 and 8 mm) and water (4 mm), which presented mean relative amounts of contrast of 0.64, 0.63 and 0.62, respectively (Table 1).

**Table 1:** Means (standard deviations) of the relative contrast of the four tested materials at the 11 thicknesses

**Thickness (mm)**	**Materials**			
	**Acrylic**	**Wood**	** Utility wax**	**Water**
				
4	0.57 (0.02) A ab	0.57 (0.05) A a	0.64 (0.02) A a *	0.62 (0.01) A a *
8	0.59 (0.03) A a	0.57 (0.06) A a	0.63 (0.01) A a *	0.59 (0.01) A ab
12	0.51 (0.02) A abc	0.58 (0.07) A a	0.55 (0.06) A ab	0.54 (0.00) A abc
15	0.47 (0.02) A abcde	0.52 (0.02) A ab	0.59 (0.08) A ab	0.53 (0.00) A abcd
20	0.44 (0.00) A bcde	0.55 (0.02) A ab	0.55 (0.02) A abc	0.45 (0.01) A bcd
24	0.46 (0.00) A abcde	0.49 (0.04) A ab	0.53 (0.11) A abc	0.47 (0.01) A bcd
28	0.48 (0.02) A abcde	0.42 (0.01) A b	0.55 (0.01) A abc	0.49 (0.01) A abcd
32	0.47 (0.06) A abcde	0.51 (0.03) A ab	0.50 (0.02) A abc	0.47 (0.01) A bcd
36	0.43 (0.04) A cde	0.43 (0.04) A ab	0.47 (0.03) A bc	0.47 (0.01) A bcd
40	0.42 (0.02) A de	0.51 (0.02) A ab	0.46 (0.06) A bc	0.44 (0.02) A cd
45	0.36 (0.05) A e	0.46 (0.04) A b	0.42 (0.12) A c	0.40 (0.01) A d

Means followed by different lowercase letters in columns and uppercase letters in rows differ statistically among them (Dunnett's test; p<0.05).

* Statistically different from the patient by the Dunnett's test

When analyzing the four different materials at each specific thickness (4, 8, 12, 15, 20, 24, 28, 32, 36, 40, 45 mm), there was no statistically significant difference among them. However, when comparing one specific material at its eleven different thicknesses, there were statistically significant differences among some of the thicknesses (Table 1).

## DISCUSSION

As it is impossible to measure doses within the patient, many tissue-equivalent materials have been developed, and dosimetric studies have been conducted in phantoms that approximate the human form to reflect the x-ray absorption and scattering properties of various tissues^4^. The materials at the specific thicknesses evaluated in the present study are ideally appropriate for radiographic, dosimetric and radiobiological studies.

Water was the first soft tissue substitute material to be used in radiation measures and up to now it continues to be tested^5^. Blake, et al.^1^ (1992) studied the effects of beam hardening on measurements made with a commercial dual energy x-ray scanner. Bone was represented by layers of aluminum of linearly increasing thickness, which were scanned under water thicknesses ranging from 0 to 25 mm to represent different body thicknesses of soft tissue. Borg, et al.^2^ (1998) placed jaw specimens immediately behind a polymethylmethacrylate cylinder filled with 20 mm of water to simulate the soft tissue of the face. In the present study, a 2-mm-thick polymethylmethacrylate box filled with water was used. No significant difference was found between 8, 12, 15, 20, 24, 28, 32, 36, 40, 45 mm of water and the patient’s tissues.

Meurer^11^ (2003) testing samples of different soft tissue substitute materials to study the optical values of the human jaw, advocated that 20 mm of acrylic was the material that best reproduced the results found with the muscular tissue, used as gold standard. Gegler, et al.^8^ (2006) joined a 20-mm- thick acrylic block to a maxilla simulator model in order to simulate the soft tissue of the face. The acrylic used in this research ranged from 4 to 45 mm thick. However, none of the thicknesses tested presented statistical difference when compared with the patient, indicating that 4, 8, 12, 15, 20, 24, 28, 32, 36, 40 and 45 mm of acrylic can be used as soft tissue substitute of the face.

Brand, et al.^4^ (1989) constructed a phantom to obtain accurate estimates of radiation doses in the head and neck region. The soft tissues of the head and neck were represented by a mixture of wax, plastic, magnesium oxide, and titanium dioxide with the x-ray absorption and scattering properties close to those of water and soft tissue. Soft tissue thicknesses were based on depths reported in the literature and supplemented by cadaver measurements. Conversely, the soft-tissueequivalent material used in this study was utility wax alone, due to its availability and being easy to use. The wax thickness ranged from 4 to 45 mm, and only two values differed statistically from those of the patient’s tissues: utility wax 4 mm and 8 mm, which presented mean relative amounts of contrast of 0.64 and 0.63, respectively.

Demann, et al.^6^ (2002) investigated the effects of soft tissue and position on vector during distraction. The authors used polyethylene straps in the temporomandibular joint region in a manner that resembled the origin and insertion of the masticatory muscles. They concluded that simulated soft tissues of the face affected the vector of distraction. Other materials, such as epoxy resin and hydrophilic materials, have also been used to substitute soft tissue in Oral Radiology^7,14^.

In this study, the materials were chosen according to their availability and reproducibility. The thicknesses of the materials were determined based on the reference values of the patients. Thus eleven different thicknesses were established for each material in order to find out the specific thicknesses that presented no statistical difference from those of the patients. The contrast values of the materials tested resemble human biological tissue.

Phantom materials are used to simulate the interactions of electromagnetic radiation with the body tissue and organs. A material that scatters and absorbs radiation in a similar way as that of the body is a potentially useful phantom material^7^. However, there are very few reports in the literature concerning materials used as soft tissue substitutes in Oral Radiology research. In this study, different possibilities of materials and thicknesses that can be used to replace soft tissues in Dentistry have been presented.

The clinical significance of the present work is related to the fact that the reference values were obtained from patients with different tissue densities. Therefore, the patient’s muscle used as gold standard was more precise and reliable.

## CONCLUSION

It was possible to conclude that except for utility wax (4 mm and 8 mm) and water (4 mm), all materials tested at different thickness could be used to simulate soft tissues in Oral Radiology studies.
